# Supplementary material for: Breast Reconstruction Use and Impact on Surgical and Oncologic Outcomes Amongst Inflammatory Breast Cancer Patients—A Systematic Review
Source: Curr Oncol. 2023 Jul 13;30(7):6666–81. doi: 10.3390/curroncol30070489 (PMC10377939; doi:10.3390/curroncol30070489)
Supplement: Supplementary file 1 [file curroncol-30-00489-s001.zip › curroncol-2430262-supplementary.pdf]

SUPPLEMENTARY MATERIALS

Table S1: Search Strategy

MEDLINE	EMBASE	Cochrane Library
1. inflammatory	1. inflammatory	1. MeSH descriptor: [Inflammatory Breast Neoplasms] explode all trees
2. breast cancer	2. breast cancer	2. MeSH descriptor: [Mammaplasty] explode all trees
3. (breast neoplasm or breast neoplasms or breast malignancy or breast malignancies).mp.	3. (breast neoplasm or breast neoplasms or breast malignancy or breast malignancies).mp.	3. #1 and #2
4. 2 or 3	4. 2 or 3	4. Inflammatory breast cancer
5. 1 and 4	5. 1 and 4	5. Inflammatory breast neoplasm
6. breast reconstruction	6. breast reconstruction	6. Inflammatory breast malignancy
7. (mammoplasty or mammaplasty or mammoplasties or mammaplasties).mp.	7. (mammoplasty or mammaplasty or mammoplasties or mammaplasties).mp.	7. IBC
8. (esthetic surgery or esthetic surgeries or esthetic procedure or esthetic procedures or cosmetic surgery or cosmetic surgeries or cosmetic procedure or cosmetic procedures).mp.	8. (esthetic surgery or esthetic surgeries or esthetic procedure or esthetic procedures or cosmetic surgery or cosmetic surgeries or cosmetic procedure or cosmetic procedures).mp.	8. #4 or #5 or #6 or #7
9. 6 or 7 or 8	9. 6 or 7 or 8	9. Breast reconstruction
10. 5 and 9	10. 5 and 9	10. Mammoplasty
		11. Mammaplasty
		12. #9 or #10 or #11
		13. #8 and #12
